# Prevalence of Adverse Childhood Experiences Among U.S. Adults — Behavioral Risk Factor Surveillance System, 2011–2020

**DOI:** 10.15585/mmwr.mm7226a2

**Published:** 2023-06-30

**Authors:** Elizabeth A. Swedo, Maria V. Aslam, Linda L. Dahlberg, Phyllis Holditch Niolon, Angie S. Guinn, Thomas R. Simon, James A. Mercy

**Affiliations:** ^1^Division of Violence Prevention, National Center for Injury Prevention and Control, CDC; ^2^Division of Injury Prevention, National Center for Injury Prevention and Control, CDC.

Adverse childhood experiences (ACEs) are defined as preventable, potentially traumatic events that occur among persons aged <18 years and are associated with numerous negative outcomes; data from 25 states indicate that ACEs are common among U.S. adults (*1*). Disparities in ACEs are often attributable to social and economic environments in which some families live (*2*,*3*). Understanding the prevalence of ACEs, stratified by sociodemographic characteristics, is essential to addressing and preventing ACEs and eliminating disparities, but population-level ACEs data collection has been sporadic (*1*). Using 2011–2020 Behavioral Risk Factor Surveillance System (BRFSS) data, CDC provides estimates of ACEs prevalence among U.S. adults in all 50 states and the District of Columbia, and by key sociodemographic characteristics. Overall, 63.9% of U.S. adults reported at least one ACE; 17.3% reported four or more ACEs. Experiencing four or more ACEs was most common among females (19.2%), adults aged 25–34 years (25.2%), non-Hispanic American Indian or Alaska Native (AI/AN) adults (32.4%), non-Hispanic multiracial adults (31.5%), adults with less than a high school education (20.5%), and those who were unemployed (25.8%) or unable to work (28.8%). Prevalence of experiencing four or more ACEs varied substantially across jurisdictions, from 11.9% (New Jersey) to 22.7% (Oregon). Patterns in prevalence of individual and total number of ACEs varied by jurisdiction and sociodemographic characteristics, reinforcing the importance of jurisdiction and local collection of ACEs data to guide targeted prevention and decrease inequities. CDC has released prevention resources, including Preventing Adverse Childhood Experiences: Leveraging the Best Available Evidenc*e*, to help provide jurisdictions and communities with the best available strategies to prevent violence and other ACEs, including guidance on how to implement those strategies for maximum impact (*4*–*6*).

 BRFSS is an annual survey of health-related risk behaviors and chronic health conditions representative of noninstitutionalized adults collected from all 50 states, the District of Columbia, and three U.S. territories (*7*). In addition to core questions administered annually to all participants, jurisdictions and territories can include jurisdiction-approved optional modules, as well as jurisdiction-added questions.* From 2011 to 2020, ACEs questions were included in the BRFSS questionnaire at least once by all 50 states and the District of Columbia as either an optional module (2011–2012 and 2019–2020) or jurisdiction-added questions (2013–2018). For jurisdictions that included ACEs questions in more than 1 year, the most recent year was included. 

The optional ACEs module includes 11 questions to determine exposure to eight types of ACEs: physical abuse, emotional abuse, sexual abuse, witnessing intimate partner violence, household substance abuse, household mental illness, parental separation or divorce, and incarcerated household member^†^ (*1*). The Arkansas and New Hampshire questionnaires differed from the optional ACEs module. Arkansas collapsed three sexual abuse questions into a single question, and New Hampshire omitted two of the three sexual abuse questions.^§^ The Arkansas questionnaire also combined household drug abuse and alcohol abuse questions into a single household substance abuse question.^¶^ Responses to all ACE types were dichotomized^**^; ACE scores were calculated for participants by summing affirmative responses to all eight ACE types and then categorized into zero, one, two to three, or four or more ACEs. Four or more ACEs were selected as the upper cut-off given the volume of research linking exposure to four or more ACEs with negative health and life outcomes (*1**,**2**,**8**,**9*). The New Hampshire questionnaire did not include divorce or emotional abuse questions; therefore, the maximum ACE score in New Hampshire was six. 

Participants with missing data for any type of ACE were excluded (79,797), leaving 264,882 participants (72.5% of total). Weighted prevalence estimates and 95% CIs were calculated for individual ACEs and total ACE score, by jurisdiction and by sociodemographic characteristics (sex, age group, race and ethnicity, annual household income, educational attainment, and employment status). Age-stratified jurisdictional prevalence estimates for four or more ACEs were also calculated. All analyses accounted for survey design by using recommended weights and complex survey procedures in SAS software (version 9.4; SAS Institute). This activity was reviewed by CDC and was conducted consistent with applicable federal law and CDC policy.^††^

Survey response rate ranged by jurisdiction from 30.6% (Illinois, 2017) to 67.2% (Mississippi, 2020) (Table 1). Nearly two thirds of U.S. adults (63.9%) experienced one or more ACE: 23.1% reported one; 23.5% reported two to three; and 17.3% reported four or more ACEs (Table 2). The prevalence of four or more ACEs was highest among females (19.2%), persons aged 25–34 years (25.2%), AI/AN adults (32.4%), and multiracial adults (31.5%). The prevalence of four or more ACEs was also higher among adults with household incomes <$15,000 (24.1%), those with less than a high school education (20.5%), and those who were unable to work (28.8%). Prevalence of four or more ACEs was lowest among persons aged ≥65 years (7.7%). Emotional abuse was the most reported type of ACE (34.0%), followed by parental separation or divorce (28.4%), and household substance abuse (26.5%) (Table 3). Patterns in prevalence of individual types of ACEs differed by sociodemographic characteristics. 

**TABLE 1:** Prevalence of individual adverse childhood experience types among adults, by jurisdiction — Behavioral Factor Surveillance System, United States, 2011–2020

Jurisdiction*	Survey year	Survey response rate, %	Total no., unweighted	ACE category, weighted % (95% CI)							
				Emotional^†^	Physical	Sexual^§^	Witnessed intimate partner violence	Household substance use^¶^	Household mental illness	Parental separation or divorce^†^	Incarcerated household member
Alabama	2020	42.4	**4,281**	30.9 (29.1–32.7)	19.5 (17.8–21.1)	13.6 (12.3–15.0)	18.6 (17.0–20.1)	28.2 (26.4–30.0)	18.3 (16.7–19.8)	33.8 (31.9–35.7)	10.7 (9.3–12.0)
Alaska	2015	54.2	**3,062**	42.2 (39.3–45.1)	19.4 (17.0–21.9)	16.1 (14.1–18.2)	19.5 (17.1–22.0)	32.6 (29.8–35.4)	22.8 (20.2–25.5)	30.2 (27.5–33.0)	10.2 (8.4–12.1)
Arizona	2020	50.0	**7,682**	35.3 (33.8–36.9)	26.3 (24.9–27.8)	13.8 (12.7–14.9)	17.0 (15.7–18.2)	27.9 (26.4–29.3)	17.4 (16.1–18.7)	31.9 (30.4–33.5)	10.0 (8.9–11.0)
Arkansas	2018	55.6	**4,231**	31.9 (29.7–34.2)	17.5 (15.7–19.4)	14.3 (12.7–16.0)	19.1 (17.1–21.0)	26.4 (24.3–28.6)	20.4 (18.3–22.5)	35.7 (33.4–38.0)	10.1 (8.5–11.8)
California	2020	38.7	**1,485**	38.4 (35.2–41.6)	30.7 (27.6–33.8)	13.7 (11.5–16.0)	20.6 (17.9–23.3)	26.8 (23.9–29.8)	16.2 (13.8–18.6)	28.3 (25.4–31.2)	9.3 (7.1–11.4)
Colorado	2014	57.0	**3,553**	34.2 (32.1–36.3)	18.0 (16.2–19.7)	10.4 (9.1–11.7)	16.4 (14.7–18.0)	27.8 (25.9–29.8)	17.1 (15.5–18.7)	28.9 (26.9–31.0)	6.0 (4.9–7.2)
Connecticut	2017	37.1	**8,121**	32.5 (31.0–34.0)	15.5 (14.4–16.7)	9.1 (8.2–9.9)	13.5 (12.4–14.6)	26.0 (24.6–27.4)	15.1 (14.0–16.3)	23.8 (22.4–25.2)	6.5 (5.7–7.4)
Delaware	2019	38.2	**2,937**	35.5 (33.0–38.0)	29.2 (26.8–31.5)	12.0 (10.4–13.7)	18.0 (15.9–20.1)	27.7 (25.2–30.2)	17.2 (15.0–19.3)	27.4 (25.0–29.8)	8.5 (6.8–10.2)
District of Columbia	2020	45.1	**2,563**	36.2 (33.6–38.7)	21.0 (18.9–23.1)	12.7 (10.9–14.6)	14.4 (12.6–16.3)	21.8 (19.7–23.9)	18.2 (16.1–20.2)	33.2 (30.6–35.8)	9.4 (7.6–11.2)
Florida	2020	40.1	**7,928**	30.3 (28.0–32.7)	23.5 (21.4–25.5)	13.0 (11.2–14.7)	16.6 (14.8–18.4)	26.3 (24.0–28.5)	13.2 (11.7–14.7)	33.0 (30.5–35.5)	9.4 (7.8–11.0)
Georgia	2020	39.1	**6,595**	32.3 (30.4–34.2)	22.2 (20.4–23.9)	13.2 (11.8–14.5)	16.7 (15.2–18.1)	24.9 (23.2–26.7)	15.1 (13.6–16.5)	32.4 (30.5–34.3)	9.9 (8.6–11.3)
Hawaii	2020	42.0	**6,627**	34.0 (32.5–35.6)	25.5 (24.1–26.9)	10.8 (9.8–11.7)	17.5 (16.3–18.7)	23.5 (22.1–24.8)	13.4 (12.4–14.5)	26.3 (24.9–27.8)	9.4 (8.4–10.4)
Idaho	2020	51.0	**4,725**	36.9 (34.9–38.9)	22.9 (21.2–24.7)	13.5 (12.1–14.9)	15.5 (13.9–17.0)	28.5 (26.6–30.4)	20.9 (19.2–22.6)	30.0 (28.0–31.9)	11.7 (10.3–13.1)
Illinois	2017	30.6	**4,322**	33.8 (32.0–35.7)	16.6 (15.1–18.1)	10.8 (9.6–12.1)	16.9 (15.4–18.3)	26.6 (24.8–28.3)	16.1 (14.6–17.6)	24.0 (22.3–25.7)	7.5 (6.4–8.7)
Indiana	2019	46.2	**6,998**	35.8 (34.3–37.3)	25.2 (23.9–26.5)	14.1 (13.0–15.2)	17.9 (16.7–19.1)	26.9 (25.5–28.3)	19.9 (18.6–21.2)	30.2 (28.8–31.7)	9.6 (8.6–10.6)
Iowa	2020	55.5	**7,700**	34.9 (33.6–36.2)	21.2 (20.1–22.3)	11.9 (11.0–12.9)	16.0 (14.9–17.0)	25.0 (23.8–26.2)	19.4 (18.3–20.6)	25.0 (23.8–26.2)	7.8 (7.0–8.6)
Kansas	2020	57.8	**4,267**	35.9 (34.1–37.8)	22.8 (21.2–24.5)	13.5 (12.2–14.8)	16.4 (15.0–17.9)	27.1 (25.3–28.8)	21.8 (20.1–23.4)	29.3 (27.5–31.1)	8.3 (7.1–9.4)
Kentucky	2020	43.3	**3,101**	32.3 (30.2–34.4)	21.0 (19.2–22.8)	14.3 (12.8–15.8)	17.4 (15.7–19.1)	29.5 (27.5–31.6)	22.1 (20.2–24.0)	31.3 (29.2–33.4)	12.6 (11.1–14.2)
Louisiana	2016	30.7	**4,106**	30.7 (28.4–33.0)	14.6 (12.7–16.4)	12.0 (10.4–13.5)	20.4 (18.4–22.4)	28.0 (25.8–30.2)	17.0 (15.1–18.8)	34.2 (31.8–36.7)	10.6 (9.0–12.3)
Maine	2011	54.7	**3,555**	35.6 (33.2–38.0)	17.5 (15.7–19.4)	14.2 (12.5–15.9)	14.8 (13.1–16.6)	33.9 (31.5–36.2)	20.4 (18.2–22.6)	25.3 (23.0–27.6)	7.3 (5.7–8.9)
Maryland	2020	45.8	**3,678**	30.2 (28.1–32.4)	22.0 (20.1–24.0)	11.0 (9.6–12.4)	15.3 (13.7–17.0)	22.9 (21.0–24.9)	15.3 (13.6–17.0)	28.6 (26.4–30.7)	6.9 (5.7–8.1)
Massachusetts	2020	48.8	**2,452**	34.0 (31.5–36.5)	23.3 (21.0–25.5)	10.5 (8.9–12.0)	15.1 (13.1–17.1)	26.0 (23.7–28.3)	18.5 (16.5–20.5)	25.5 (23.2–27.8)	6.8 (5.3–8.2)
Michigan	2019	51.5	**8,900**	37.9 (36.5–39.3)	25.5 (24.3–26.8)	14.3 (13.3–15.3)	17.8 (16.7–18.9)	30.6 (29.3–31.9)	20.9 (19.7–22.1)	29.6 (28.3–30.9)	9.8 (8.8–10.8)
Minnesota	2011	51.9	**9,004**	33.8 (32.1–35.6)	15.2 (13.8–16.5)	9.5 (8.4–10.6)	14.2 (12.9–15.5)	26.7 (25.0–28.3)	15.3 (13.9–16.6)	19.2 (17.7–20.8)	6.5 (5.4–7.7)
Mississippi	2020	67.2	**5,673**	23.3 (21.7–24.9)	12.5 (11.3–13.6)	11.2 (10.0–12.3)	15.0 (13.8–16.3)	25.5 (23.9–27.0)	15.5 (14.1–16.9)	33.5 (31.8–35.3)	10.5 (9.3–11.7)
Missouri	2020	57.8	**7,672**	34.2 (32.7–35.6)	20.9 (19.7–22.2)	13.2 (12.2–14.2)	16.5 (15.4–17.7)	29.4 (28.1–30.8)	23.2 (21.9–24.5)	31.6 (30.2–33.0)	11.3 (10.3–12.3)
Montana	2020	50.4	**5,311**	38.9 (37.2–40.5)	24.6 (23.1–26.1)	14.2 (13.1–15.4)	18.1 (16.7–19.4)	34.3 (32.7–35.9)	24.6 (23.1–26.1)	31.1 (29.5–32.7)	10.6 (9.5–11.8)
Nebraska	2011	60.9	**9,288**	33.7 (31.8–35.6)	15.0 (13.6–16.5)	9.0 (8.0–10.1)	13.7 (12.4–15.1)	24.7 (23.0–26.5)	15.0 (13.5–16.5)	19.1 (17.4–20.8)	6.0 (4.8–7.1)
Nevada	2020	47.9	**1,659**	38.5 (35.2–41.8)	28.0 (24.9–31.0)	15.0 (12.6–17.4)	22.0 (19.0–25.1)	32.7 (29.5–35.9)	17.9 (15.4–20.3)	35.2 (31.9–38.4)	10.4 (8.2–12.6)
New Hampshire	2016	42.2	**5,515**	Not asked	14.9 (13.6–16.3)	11.0 (9.8–12.1)	18.0 (16.5–19.6)	29.6 (27.7–31.4)	19.1 (17.4–20.7)	Not asked	5.6 (4.5–6.6)
New Jersey	2020	34.5	**2,733**	33.4 (31.0–35.7)	24.9 (22.8–27.1)	8.6 (7.2–10.0)	14.8 (13.0–16.6)	19.7 (17.9–21.5)	13.4 (11.8–15.1)	21.7 (19.8–23.7)	5.2 (4.1–6.3)
New Mexico	2019	52.2	**4,951**	36.0 (34.1–37.9)	28.7 (26.9–30.6)	16.7 (15.2–18.2)	20.0 (18.3–21.6)	31.9 (30.1–33.8)	20.8 (19.1–22.4)	29.5 (27.6–31.4)	8.8 (7.6–10)
New York	2019	37.3	**3,571**	31.2 (29.1–33.4)	24.9 (22.9–26.9)	11.1 (9.6–12.6)	14.9 (13.3–16.6)	20.3 (18.4–22.1)	14.9 (13.1–16.6)	24.6 (22.6–26.7)	4.8 (3.7–5.9)
North Carolina	2014	37.5	**2,913**	28.1 (25.6–30.5)	13.5 (11.7–15.4)	11.9 (10.1–13.7)	16.5 (14.6–18.5)	27.4 (25.0–29.8)	15.2 (13.1–17.3)	29.7 (27.1–32.3)	7.0 (5.6–8.4)
North Dakota	2020	55.6	**3,790**	34.0 (31.8–36.2)	20.7 (18.8–22.6)	11.1 (9.6–12.7)	13.5 (11.9–15.1)	27.9 (25.8–30.0)	18.2 (16.3–20.1)	23.0 (21.0–25.1)	8.1 (6.7–9.5)
Ohio	2019	46.4	**7,366**	38.2 (36.4–40.1)	24.8 (23.1–26.4)	12.9 (11.7–14.2)	17.3 (15.9–18.8)	27.6 (25.9–29.2)	20.3 (18.7–21.9)	31.6 (29.8–33.5)	10.9 (9.5–12.3)
Oklahoma	2020	52.4	**2,029**	30.0 (27.4–32.7)	19.4 (17.2–21.5)	12.8 (11.0–14.7)	17.2 (15.1–19.4)	28.2 (25.6–30.7)	19.8 (17.6–22.0)	34.2 (31.5–37.0)	10.7 (8.8–12.6)
Oregon	2018	39.8	**2,969**	40.0 (34.6–45.4)	22.2 (18.2–26.2)	18.0 (16.0–20.0)	19.4 (15.8–23.0)	32.3 (28.7–36.0)	23.9 (20.3–27.4)	32.7 (25.0–40.5)	10.0 (6.3–13.7)
Pennsylvania	2019	46.6	**5,219**	36.0 (34.3–37.7)	25.5 (23.9–27.0)	11.8 (10.7–12.9)	16.7 (15.4–18.1)	28.0 (26.4–29.6)	19.2 (17.8–20.6)	26.8 (25.2–28.4)	9.7 (8.6–10.8)
Rhode Island	2020	39.1	**4,235**	34.1 (31.9–36.3)	24.0 (22.0–25.9)	10.1 (8.8–11.3)	15.3 (13.7–17.0)	25.6 (23.6–27.6)	18.9 (17.0–20.8)	29.5 (27.3–31.6)	6.6 (5.3–7.9)
South Carolina	2020	47.9	**2,987**	31.3 (29.1–33.4)	21.3 (19.5–23.2)	14.8 (13.1–16.4)	15.2 (13.6–16.8)	29.3 (27.2–31.5)	19.0 (17.1–20.8)	30.6 (28.4–32.8)	10.3 (8.8–11.7)
South Dakota	2020	61.2	**5,584**	34.2 (31.3–37.1)	20.4 (18.0–22.7)	10.5 (8.7–12.3)	12.2 (10.4–14.1)	25.5 (22.8–28.1)	16.2 (13.8–18.7)	25.9 (23.1–28.7)	7.7 (6.0–9.4)
Tennessee	2019	42.0	**4,508**	34.5 (32.5–36.5)	23.9 (22.1–25.7)	15.8 (14.2–17.3)	18.9 (17.2–20.5)	31.4 (29.4–33.4)	20.5 (18.8–22.2)	34.1 (32.0–36.1)	10.7 (9.2–12.2)
Texas	2020	40.6	**7,603**	30.9 (28.8–33.0)	26.5 (24.5–28.6)	12.3 (10.9–13.7)	17.8 (16.0–19.5)	23.7 (21.7–25.6)	13.8 (12.3–15.3)	28.4 (26.4–30.4)	7.9 (6.8–9.1)
Utah	2020	55.4	**9,155**	42.3 (40.9–43.6)	27.6 (26.4–28.8)	15.5 (14.5–16.5)	17.1 (16.1–18.2)	25.3 (24.1–26.5)	28.1 (26.9–29.3)	24.0 (22.8–25.2)	9.1 (8.3–9.9)
Vermont	2011	49.9	**5,960**	33.0 (31.2–34.8)	14.7 (13.4–16.1)	10.4 (9.3–11.5)	14.4 (13.1–15.7)	28.1 (26.3–29.8)	17.2 (15.7–18.7)	23.1 (21.4–24.8)	5.6 (4.4–6.8)
Virginia	2020	41.5	**7,167**	32.6 (31.0–34.2)	21.9 (20.6–23.3)	11.0 (10.0–12.0)	15.5 (14.3–16.7)	23.3 (21.9–24.7)	15.7 (14.5–17.0)	27.7 (26.2–29.2)	8.1 (7.1–9.1)
Washington	2011	44.3	**12,798**	40.1 (38.6–41.5)	19.9 (18.7–21.1)	14.7 (13.7–15.7)	19.5 (18.3–20.7)	31.2 (29.9–32.6)	20.5 (19.3–21.7)	28.2 (26.8–29.5)	8.0 (7.0–8.9)
West Virginia	2019	49.6	**4,523**	29.9 (28.1–31.7)	20.1 (18.5–21.8)	13.1 (11.8–14.5)	17.8 (16.3–19.4)	27.1 (25.3–28.9)	19.4 (17.7–21.1)	26.9 (25.0–28.7)	9.0 (7.7–10.4)
Wisconsin	2020	53.8	**3,951**	38.1 (36.0–40.3)	25.6 (23.7–27.6)	12.3 (10.9–13.8)	16.6 (14.9–18.3)	27.2 (25.3–29.2)	18.0 (16.2–19.8)	23.9 (21.9–25.9)	7.6 (6.3–8.9)
Wyoming	2020	55.9	**3,879**	36.5 (34.2–38.8)	24.9 (22.8–27.0)	11.8 (10.3–13.4)	17.2 (15.3–19.1)	29.5 (27.3–31.7)	20.1 (18.1–22.2)	30.9 (28.7–33.2)	11.2 (9.4–13.0)

**Abbreviation:** ACE = adverse childhood experience.

* For jurisdictions that included ACE questions in >1 year, the most recent year was included.

^†^ New Hampshire did not include these questions on its survey.

^§^ Arkansas collapsed three sexual abuse questions into a single question; New Hampshire omitted two of the three sexual abuse questions. Arkansas’ sexual abuse question was worded, “How often did anyone at least 5 years older than you or an adult ever touch you sexually, try to make you touch them sexually, or force you to have sex?” New Hampshire only included one of the three sexual abuse questions, “How often did anyone at least 5 years older than you or an adult ever touch you sexually?”

^¶^ The Arkansas questionnaire combined household drug abuse and alcohol abuse questions into a single household substance abuse question, “Did you live with anyone who was a problem drinker or alcoholic, or who used illegal street drugs or abused prescription medications?”

**TABLE 2:** Adverse childhood experiences scores among adults, by sociodemographic characteristics and jurisdiction — Behavioral Risk Factor Surveillance System, United States, 2011–2020

Characteristic	Total no.*, unweighted	ACE score, weighted % (95% CI)			
		0	1	2**–**3	≥4
**Total**	**264,882**	**36.1 (35.6**–**36.6)**	**23.1 (22.7**–**23.6)**	**23.5 (23.0**–**23.9)**	**17.3 (16.9**–**17.7)**
**Sex (missing = 20)**					
Female	**149,565**	36.0 (35.3–36.7)	22.1 (21.5–22.7)	22.7 (22.1–23.4)	19.2 (18.6–19.8)
Male	**115,297**	36.3 (35.5–37.0)	24.2 (23.6–24.9)	24.2 (23.5–25.0)	15.2 (14.6–15.9)
**Age group, yrs (missing **= **2,961)**					
18**–**24	**13,483**	28.9 (27.2–30.6)	23.1 (21.6–24.7)	25.9 (24.2–27.6)	22.1 (20.6–23.6)
25**–**34	**23,731**	27.3 (26.1–28.6)	22.2 (21.0–23.3)	25.3 (23.9–26.7)	25.2 (23.8–26.5)
35**–**44	**31,113**	32.8 (31.3–34.3)	21.9 (20.7–23.2)	24.8 (23.5–26.1)	20.5 (19.3–21.7)
45**–**54	**40,962**	34.1 (32.8–35.3)	23.2 (22.1–24.3)	24.1 (23.0–25.3)	18.6 (17.5–19.7)
55**–**64	**55,571**	37.5 (36.4–38.7)	23.7 (22.8–24.7)	24.1 (23.1–25.2)	14.6 (13.8–15.4)
≥65	**97,061**	49.3 (48.3–50.4)	24.1 (23.2–25.0)	18.8 (18.1–19.6)	7.7 (7.1–8.2)
**Race and ethnicity (missing = 6,940)**					
AI/AN, non-Hispanic	**4,256**	25.4 (21.8–29.0)	17.6 (14.9–20.3)	24.6 (21.1–28.1)	32.4 (27.7–37.2)
Asian, non-Hispanic	**5,199**	49.8 (45.4–54.3)	23.0 (19.0–27.0)	18.8 (15.2–22.4)	8.3 (5.9–10.8)
Black or African American, non-Hispanic	**18,558**	29.9 (28.5–31.2)	26.0 (24.6–27.4)	26.1 (24.6–27.5)	18.1 (16.9–19.2)
NH/OPI, non-Hispanic	**876**	33.3 (24.7–42.0)	20.4 (14.6–26.3)	23.0 (16.9–29.2)	23.2 (13.6–32.9)
White, non-Hispanic	**205,306**	37.1 (36.5–37.6)	23.1 (22.6–23.5)	23.1 (22.6–23.5)	16.8 (16.4–17.3)
Hispanic or Latino	**16,995**	34.9 (33.0–36.8)	22.6 (21.1–24.1)	23.9 (22.2–25.6)	18.6 (17.1–20.1)
Multiracial, non-Hispanic	**5,105**	22.9 (19.0–26.8)	16.2 (13.8–18.5)	29.5 (25.1–33.9)	31.5 (27.4–35.5)
Other race, non-Hispanic	**1,647**	28.5 (22.9–34.1)	21.5 (16.6–26.3)	26.8 (18.8–34.7)	23.3 (17.3–29.3)
**Household income USD (missing = 39,409)**					
<$15,000	**18,902**	31.6 (29.4–33.7)	19.8 (18.3–21.4)	24.5 (22.5–26.6)	24.1 (22.3–25.9)
$15,000**–**$24,999	**34,874**	33.1 (31.8–34.4)	22.5 (21.3–23.7)	22.5 (21.3–23.6)	21.9 (20.8–23.1)
$25,000**–**$34,999	**23,665**	31.9 (30.4–33.5)	23.8 (22.3–25.2)	24.3 (22.6–26.0)	20.0 (18.5–21.5)
$35,000**–**$49,999	**32,252**	34.9 (33.4–36.4)	23.6 (22.2–24.9)	22.6 (21.5–23.8)	18.9 (17.6–20.1)
≥$50,000	**115,780**	36.8 (35.9–37.6)	23.5 (22.8–24.2)	24.5 (23.7–25.2)	15.3 (14.6–15.9)
**Education level (missing = 584)**					
Less than high school diploma	**16,944**	35.2 (33.4-37.1)	22.9 (21.3-24.4)	21.4 (19.8-23.0)	20.5 (19.0-22.0)
High school diploma or GED	**71,799**	35.3 (34.3–36.3)	23.7 (22.8–24.6)	22.5 (21.7–23.4)	18.4 (17.5–19.3)
Some college	**74,362**	32.3 (31.4–33.2)	22.4 (21.6–23.2)	25.5 (24.6–26.5)	19.8 (19.0–20.6)
College degree	**101,193**	41.2 (40.4–42.1)	23.5 (22.7–24.2)	23.1 (22.3–23.8)	12.2 (11.6–12.8)
**Employment status (missing = 1,484)**					
Employed	**130,794**	34.0 (33.3–34.7)	23.7 (23.1–24.3)	24.3 (23.7–24.9)	18.0 (17.4–18.6)
Unemployed	**12,470**	25.6 (23.3–27.8)	20.9 (18.9–22.8)	27.7 (25.4–30.1)	25.8 (23.6–28.0)
Unable to work	**17,833**	26.0 (24.3–27.7)	19.6 (18.3–20.9)	25.6 (23.8–27.3)	28.8 (27.1–30.4)
Other	**102,301**	44.3 (43.3–45.2)	23.4 (22.5–24.2)	20.5 (19.7–21.4)	11.8 (11.1–12.6)
**Jurisdiction**					
Alabama	**4,281**	36.0 (34.2–37.9)	23.6 (21.9–25.3)	21.7 (20.1–23.3)	18.7 (17.0–20.3)
Alaska	**3,062**	31.9 (29.3–34.5)	22.2 (19.7–24.7)	23.5 (21.1–25.8)	22.3 (19.7–25.0)
Arizona	**7,682**	33.2 (31.6–34.7)	23.2 (21.9–24.6)	24.8 (23.3–26.2)	18.9 (17.6–20.2)
Arkansas^†^	**4,231**	36.4 (34.3–38.4)	23.1 (21.0–25.1)	21.0 (19.1–22.9)	19.6 (17.6–21.6)
California	**1,485**	31.7 (28.7–34.8)	20.9 (18.2–23.5)	28.5 (25.5–31.4)	19.0 (16.3–21.6)
Colorado	**3,553**	36.9 (34.9–39.0)	24.1 (22.3–26.0)	23.8 (21.8–25.7)	15.1 (13.6–16.7)
Connecticut	**8,121**	40.2 (38.8–41.7)	24.1 (22.8–25.5)	22.6 (21.3–24.0)	13.0 (11.9–14.1)
Delaware	**2,937**	33.2 (30.8–35.6)	23.5 (21.3–25.7)	25.4 (23.0–27.7)	17.9 (15.8–20.1)
District of Columbia	**2,563**	31.1 (28.7–33.5)	26.0 (23.7–28.4)	27.7 (25.3–30.1)	15.2 (13.2–17.1)
Florida	**7,928**	37.3 (34.9–39.8)	23.4 (21.2–25.5)	22.2 (20.1–24.4)	17.1 (15.2–18.9)
Georgia	**6,595**	35.3 (33.5–37.2)	24.6 (22.8–26.3)	23.4 (21.6–25.1)	16.7 (15.2–18.3)
Hawaii	**6,627**	37.3 (35.7–38.9)	23.6 (22.2–25.0)	23.6 (22.3–25.0)	15.4 (14.3–16.6)
Idaho	**4,725**	35.3 (33.3–37.2)	22.7 (21.1–24.4)	22.5 (20.8–24.2)	19.5 (17.8–21.3)
Illinois	**4,322**	41.2 (39.3–43.1)	22.8 (21.2–24.4)	19.7 (18.1–21.3)	16.4 (14.9–17.9)
Indiana	**6,998**	34.2 (32.9–35.6)	23.5 (22.2–24.8)	23.2 (21.9–24.5)	19.1 (17.8–20.3)
Iowa	**7,700**	39.4 (38.1–40.6)	22.9 (21.8–24.0)	21.1 (20.0–22.2)	16.6 (15.5–17.7)
Kansas	**4,267**	35.1 (33.4–36.9)	23.6 (21.9–25.2)	22.9 (21.2–24.5)	18.4 (16.9–20.0)
Kentucky	**3,101**	35.9 (33.8–38.0)	22.0 (20.1–23.9)	23.0 (21.1–24.9)	19.1 (17.3–20.9)
Louisiana	**4,106**	36.0 (33.7–38.3)	23.1 (21.0–25.2)	23.4 (21.3–25.6)	17.5 (15.6–19.4)
Maine	**3,555**	36.9 (34.6–39.2)	22.5 (20.3–24.6)	22.1 (20.2–24.1)	18.5 (16.4–20.5)
Maryland	**3,678**	38.1 (35.8–40.4)	24.2 (22.2–26.2)	22.7 (20.8–24.6)	15.0 (13.4–16.7)
Massachusetts	**2,452**	38.5 (36.0–41.0)	21.5 (19.4–23.6)	23.8 (21.5–26.0)	16.2 (14.2–18.2)
Michigan	**8,900**	31.7 (30.4–32.9)	23.8 (22.7–25.0)	24.7 (23.5–25.9)	19.8 (18.6–21.0)
Minnesota	**9,004**	42.0 (40.2–43.8)	23.2 (21.6–24.7)	21.6 (20.1–23.1)	13.2 (11.9–14.6)
Mississippi	**5,673**	40.4 (38.8–42.1)	25.8 (24.2–27.3)	19.1 (17.7–20.5)	14.7 (13.4–16.1)
Missouri	**7,672**	35.1 (33.7–36.5)	23.2 (21.9–24.4)	21.7 (20.5–23.0)	20.0 (18.7–21.2)
Montana	**5,311**	31.8 (30.3–33.2)	22.1 (20.8–23.5)	23.8 (22.4–25.2)	22.3 (20.8–23.8)
Nebraska	**9,288**	44.0 (42.1–45.9)	22.9 (21.3–24.6)	20.7 (19.1–22.3)	12.4 (11.0–13.8)
Nevada	**1,659**	30.2 (27.0–33.4)	21.0 (18.2–23.7)	26.8 (23.7–29.9)	22.1 (19.3–24.9)
New Hampshire^†^	**5,515**	52.6 (50.6–54.5)	22.0 (20.4–23.6)	17.8 (16.3–19.3)	7.6 (6.5–8.8)
New Jersey	**2,733**	38.3 (35.9–40.8)	25.5 (23.4–27.7)	24.3 (22.2–26.3)	11.9 (10.3–13.4)
New Mexico	**4,951**	32.0 (30.2–33.9)	22.3 (20.6–23.9)	24.6 (22.9–26.3)	21.1 (19.4–22.8)
New York	**3,571**	39.1 (36.7–41.4)	24.4 (22.4–26.4)	23.1 (21.1–25.0)	13.5 (11.9–15.1)
North Carolina	**2,913**	39.8 (37.3–42.3)	24.7 (22.4–27.0)	20.3 (18.1–22.5)	15.2 (13.2–17.3)
North Dakota	**3,790**	40.9 (38.8–43.0)	22.4 (20.6–24.2)	20.3 (18.4–22.1)	16.4 (14.6–18.2)
Ohio	**7,366**	32.4 (30.7–34.0)	22.8 (21.2–24.4)	26.1 (24.4–27.7)	18.8 (17.2–20.3)
Oklahoma	**2,029**	38.6 (35.9–41.4)	22.5 (20.1–24.9)	19.4 (17.2–21.5)	19.5 (17.2–21.8)
Oregon	**2,969**	31.5 (26.7–36.2)	22.5 (20.1–24.8)	23.4 (21.7–25.0)	22.7 (17.2–28.2)
Pennsylvania	**5,219**	35.9 (34.2–37.6)	22.4 (21.0–23.9)	22.8 (21.3–24.3)	18.9 (17.5–20.3)
Rhode Island	**4,235**	36.5 (34.3–38.6)	24.2 (22.2–26.1)	23.1 (21.2–25.0)	16.3 (14.5–18.0)
South Carolina	**2,987**	35.3 (33.1–37.5)	25.1 (23.0–27.2)	21.2 (19.3–23.1)	18.4 (16.5–20.2)
South Dakota	**5,584**	39.7 (37.0–42.4)	23.3 (20.7–25.8)	22.2 (19.6–24.9)	14.8 (12.6–16.9)
Tennessee	**4,508**	33.2 (31.3–35.1)	21.6 (20.0–23.2)	23.4 (21.6–25.2)	21.8 (20.0–23.6)
Texas	**7,603**	37.5 (35.2–39.8)	24.0 (22.1–26.0)	22.4 (20.5–24.2)	16.1 (14.5–17.8)
Utah	**9,155**	32.2 (31.0-33.4)	21.5 (20.5–22.6)	26.1 (24.9–27.2)	20.2 (19.1–21.3)
Vermont	**5,960**	40.4 (38.6–42.2)	24.2 (22.5–25.8)	20.9 (19.4–22.4)	14.5 (13.1–16.0)
Virginia	**7,167**	38.3 (36.7–39.9)	24.5 (23.0–25.9)	21.5 (20.1–22.8)	15.8 (14.5–17.0)
Washington	**12,798**	33.1 (31.8–34.4)	22.8 (21.6–24.0)	24.6 (23.3–25.8)	19.5 (18.3–20.7)
West Virginia	**4,523**	41.6 (39.8–43.4)	20.0 (18.5–21.4)	19.8 (18.2–21.4)	18.6 (17.0–20.3)
Wisconsin	**3,951**	35.5 (33.5–37.6)	24.1 (22.2–26.0)	23.6 (21.7–25.5)	16.8 (15.1–18.5)
Wyoming	**3,879**	36.0 (33.9–38.2)	22.5 (20.6–24.4)	21.5 (19.6–23.4)	20.0 (18.0–22.0)

**Abbreviations:** ACE = adverse childhood experience; AI/AN = American Indian or Alaska Native; GED = general educational development certificate; NH/OPI = Native Hawaiian or other Pacific Islander; USD = U.S. dollars.

* For jurisdictions that included ACE questions in >1 year, the most recent year was included.

^†^ Arkansas and New Hampshire’s questionnaires differed slightly from the optional ACEs module. Arkansas collapsed three sexual abuse questions into a single question; New Hampshire omitted two of the three sexual abuse questions. Arkansas’ sexual abuse question was worded, “How often did anyone ≥5 years older than you or an adult ever touch you sexually, try to make you touch them sexually, or force you to have sex?” New Hampshire only included one of the three sexual abuse questions, “How often did anyone at least 5 years older than you or an adult ever touch you sexually?” In addition, the Arkansas questionnaire combined household drug abuse and alcohol abuse questions into a single household substance abuse question, “Did you live with anyone who was a problem drinker or alcoholic, or who used illegal street drugs or abused prescription medications?” New Hampshire omitted questions related to emotional abuse and parental separation or divorce; therefore, its maximum ACE score was 6, rather than 8.

**TABLE 3:** Prevalence of individual adverse childhood experiences among adults, by sociodemographic characteristics — Behavioral Risk Factor Surveillance System, United States, 2011–2020

Characteristic	Total no.*, unweighted	Weighted % (95% CI)	Emotional^†^	Physical	Sexual^§^	ACE category, weighted % (95% CI)				
						Witnessed intimate partner violence	Household substance use^¶^	Household mental illness	Parental separation or divorce^†^	Incarcerated household member
**Total**	**264,882**	**NA**	**NA**	**23.3 (22.8–23.8)**	**12.6 (12.2–13.0)**	**17.2 (16.8–17.7)**	**26.5 (26.0–27.0)**	**17.3 (16.9–17.7)**	**28.4 (27.9–28.9)**	**8.6 (8.3-9)**
**Sex (missing = 20)**										
Female	**149,565**	52.0 (51.4–52.5)	34.0 (33.3–34.7)	22.7 (22.0–23.3)	17.7 (17.1–18.3)	18.1 (17.5–18.7)	27.9 (27.2–28.5)	19.9 (19.4–20.5)	28.4 (27.7–29.1)	8.1 (7.6–8.5)
Male	**115,297**	47.5 (47.4–48.5)	34.0 (33.2–34.8)	24.0 (23.3–24.7)	7.0 (6.6–7.4)	16.3 (15.7–16.9)	25.0 (24.3–25.7)	14.4 (13.8–14.9)	28.4 (27.7–29.1)	9.3 (8.7–9.8)
**Age group, yrs (missing = 2,961)**										
18**–**24	**13,483**	11.9 (11.5–12.4)	43.1 (41.3–45.0)	23.0 (21.4–24.6)	11.5 (10.2–12.8)	16.9 (15.6–18.3)	27.5 (25.9–29.2)	27.0 (25.5–28.6)	36.5 (34.8–38.2)	15.4 (14.0–16.7)
25**–**34	**23,731**	16.1 (15.6–16.5)	42.5 (40.9–44.0)	25.5 (24.1–27.0)	13.1 (12.1–14.2)	21.7 (20.3–23.0)	31.9 (30.4–33.3)	25.6 (24.3–26.8)	40.2 (38.7–41.7)	15.7 (14.5–16.9)
35**–**44	**31,113**	16.0 (15.6–16.45)	36.3 (34.9–37.8)	25.3 (23.9–26.7)	13.4 (12.5–14.3)	19.2 (18.1–20.3)	28.6 (27.3–29.9)	19.5 (18.3–20.7)	35.2 (33.8–36.6)	10.2 (9.2–11.2)
45**–**54	**40,962**	16.2 (15.8–16.6)	35.7 (34.4–36.9)	25.4 (24.2–26.7)	15.9 (14.8–16.9)	19.3 (18.2–20.4)	28.3 (27.1–29.5)	16.0 (15.0–16.9)	29.1 (27.9–30.2)	6.9 (6.1–7.7)
55**–**64	**55,571**	17.1 (16.7–17.4)	31.8 (30.7–32.9)	23.9 (22.8–25.0)	12.9 (12.2–13.6)	17.1 (16.2–18.0)	26.8 (25.8–27.9)	13.5 (12.8–14.2)	22.3 (21.4–23.3)	5.2 (4.7–5.7)
≥65	**97,061**	22.1 (21.7–22.5)	21.6 (20.8–22.5)	18.5 (17.7–19.2)	9.4 (8.8–10.0)	11.3 (10.6–12.1)	18.9 (18.2–19.6)	8.0 (7.5–8.5)	14.6 (13.9–15.2)	2.6 (2.3–2.8)
**Race and ethnicity (missing = 6,940)**										
AI/AN, non-Hispanic	**4,256**	1.0 (0.9–1.1)	42.1 (37.5–46.8)	31.9 (27.2–36.7)	18.8 (16.2–21.5)	29.9 (25.3–34.4)	44.5 (39.9–49.1)	26.3 (21.3–31.2)	42.0 (37.4–46.6)	17.3 (14.5–20.1)
Asian, non-Hispanic	**5,199**	4.8 (4.4–5.2)	27.9 (23.9–31.9)	20.8 (17.1–24.4)	7.5 (5.2–9.8)	15.5 (11.6–19.3)	10.7 (7.8–13.6)	8.8 (6.3–11.3)	11.5 (9.0–14.1)	3.6 (1.5–5.7)
Black or African American, non-Hispanic	**18,558**	11.0 (10.7–11.3)	30.5 (29.1–32.0)	22.5 (21.2–23.9)	14.6 (13.5–15.6)	20.4 (19.1–21.7)	24.2 (22.9–25.4)	11.9 (10.9–12.8)	41.7 (40.2–43.3)	14.2 (13.2–15.2)
NH/OPI, non-Hispanic	**876**	0.2 (0.2–0.2)	38.8 (29.5–48.1)	30.1 (20.6–39.6)	21.2 (11.4–30.9)	27.3 (17.5–37.1)	30.4 (20.8–39.9)	17.2 (9.5–24.9)	27.3 (20.6–34.1)	10.5 (6.8–14.3)
White, non-Hispanic	**205,306**	63.2 (62.6–63.8)	34.9 (34.4–35.5)	21.4 (20.9–21.9)	12.0 (11.7–12.4)	15.3 (14.9–15.7)	27.9 (27.4–28.4)	19.5 (19.0–20.0)	26.2 (25.7–26.7)	7.5 (7.1–7.8)
Hispanic or Latino	**16,995**	15.9 (15.4–16.4)	32.2 (30.3–34.0)	30.1 (28.2–31.9)	13.3 (11.9–14.7)	21.3 (19.8–22.9)	25.2 (23.6–26.9)	12.5 (11.3–13.7)	30.7 (28.9–32.4)	9.5 (8.4–10.7)
Multiracial, non-Hispanic	**5,105**	1.4 (1.3–1.5)	48.0 (43.5–52.4)	31.5 (27.7–35.3)	21.6 (18.1–25.1)	25.4 (21.8–29.1)	37.9 (33.7–42.1)	31.6 (27.3–36.0)	40.4 (36.1–44.6)	17.5 (14.0–21.1)
Other race, non-Hispanic	**1,647**	0.4 (0.4–0.5)	42.8 (35.2–50.4)	35.6 (27.5–43.7)	16.4 (11.6–21.1)	21.8 (16.8–26.8)	26.0 (20.1–31.8)	19.0 (13.3–24.6)	29.3 (23.3–35.3)	10.1 (5.4–14.7)
**Household income USD (missing = 39,409)**										
<$15,000	**18,902**	8.2 (7.8–8.5)	35.8 (33.7–38.0)	31.0 (28.9–33.2)	19.1 (17.5–20.8)	24.8 (22.8–26.8)	30.6 (28.7–32.6)	18.6 (17.2–20.0)	33.9 (31.9–35.9)	12.4 (11.0–13.7)
$15,000**–**$24,999	**34,874**	13.1 (12.7–13.4)	34.1 (32.8–35.4)	27.3 (26–28.6)	15.5 (14.5–16.4)	21.1 (20.0–22.2)	29.8 (28.5–31.0)	17.8 (16.8–18.8)	34.2 (32.9–35.6)	11.4 (10.5–12.2)
$25,000**–**$34,999	**23,665**	8.3 (8.0–8.6)	34.5 (32.7–36.3)	26.3 (24.6–28.0)	15.3 (13.6–17.1)	20.0 (18.6–21.4)	29.3 (27.8–30.9)	18.1 (16.7–19.5)	32.8 (31.0–34.6)	10.3 (9.2–11.3)
$35,000**–**$49,999	**32,252**	11.1 (10.8–11.4)	34.5 (33.0–35.9)	23.7 (22.4–25.1)	15.3 (13.6–17.1)	17.7 (16.6–18.7)	27.4 (26.0–28.8)	18.6 (17.5–19.8)	29.1 (27.7–30.5)	10.0 (9.0–11.0)
>$50,000	**115,780**	44.9 (44.3–45.4)	35.2 (34.4–36.1)	21.3 (20.5–22.0)	10.8 (10.3–11.3)	15.6 (14.9–16.3)	25.7 (24.9–26.4)	17.4 (16.7–18.1)	25.5 (24.8–26.3)	7.1 (6.5–7.6)
**Education level (missing = 584)**										
Less than high school	**16,944**	12.3 (11.9–12.7)	29.8 (28.1–31.6)	29.2 (27.4–31.1)	14.6 (13.2–16.0)	22.3 (20.7–23.8)	29.0 (27.3–30.7)	13.3 (12.2–14.4)	32.2 (30.5–34.0)	11.9 (10.8–13.0)
High school diploma or GED	**71,799**	27.6 (27.1–28.1)	32.6 (31.6–33.6)	23.5 (22.6–24.5)	11.9 (11.3–12.6)	17.9 (17.1–18.8)	28.3 (27.3–29.2)	16.0 (15.2–16.8)	32.3 (31.3–33.3)	10.5 (9.8–11.3)
Some college	**74,362**	31.0 (30.5–31.5)	38.2 (37.2–39.2)	24.8 (23.9–25.7)	14.4 (13.7–15.2)	18.6 (17.8–19.4)	29.2 (28.3–30.1)	20.5 (19.7–21.3)	30.5 (29.6–31.4)	9.4 (8.8–10.1)
College degree	**101,193**	28.8 (28.4–29.3)	32.8 (31.9–33.6)	19.0 (18.2–19.7)	10.4 (9.9–10.9)	13.0 (12.4–13.7)	21.0 (20.4–21.7)	16.7 (16.1–17.3)	20.9 (20.1–21.6)	4.6 (4.2–5.0)
**Employment status (missing = 1,484)**										
Employed	**130,794**	55.4 (54.8–55.9)	35.7 (35.0–36.4)	23.1 (22.5–23.8)	11.6 (11.1–12.1)	17.4 (16.8–17.9)	27.5 (26.8–28.1)	18.2 (17.6–18.7)	31.0 (30.3–31.7)	9.4 (8.9–9.9)
Unable to work	**17,833**	6.5 (6.2–6.7)	40.1 (38.3–41.9)	33.1 (31.3–34.9)	23.5 (22.0–25.0)	26.0 (24.4–27.6)	37.1 (35.4–38.9)	24.2 (22.6–25.7)	37.1 (35.3–38.9)	13.8 (12.4–15.2)
Unemployed	**12,470**	6.7 (6.4–7.1)	43.8 (41.3–46.3)	32.0 (29.5–34.6)	16.7 (15.1–18.4)	24.2 (22.0–26.5)	32.4 (30.1–34.7)	23.1 (21.1–25.0)	39.0 (36.6–41.5)	15.1 (13.2–16.9)
Other	**102,301**	30.7 (30.2–31.2)	27.7 (26.7–28.6)	19.7 (18.9–20.6)	11.2 (10.4–11.9)	13.7 (12.9–14.5)	21.4 (20.6–22.2)	13.0 (12.3–13.7)	19.5 (18.8–20.3)	4.8 (4.3–5.2)

**Abbreviations:** ACE = adverse childhood experience; AI/AN = American Indian or Alaska Native; GED = general educational development certificate; NA = not applicable; NH/OPI = Native Hawaiian or other Pacific Islander; USD = U.S. dollars.

* For jurisdictions that included ACE questions in >1 year, the most recent year was included.

^†^ New Hampshire did not include these questions on its survey.

^§^ Arkansas collapsed three sexual abuse questions into a single question; New Hampshire omitted two of the three sexual abuse questions. Arkansas’ sexual abuse question was worded, “How often did anyone at least 5 years older than you or an adult ever touch you sexually, try to make you touch them sexually, or force you to have sex?” New Hampshire only included one of the three sexual abuse questions, “How often did anyone at least 5 years older than you or an adult ever touch you sexually?”

^¶^ The Arkansas questionnaire combined household drug abuse and alcohol abuse questions into a single household substance abuse question, “Did you live with anyone who was a problem drinker or alcoholic, or who used illegal street drugs or abused prescription medications?”

Prevalence of individual ACEs (Table 3), total number of ACEs (Table 1), and four or more ACEs (Supplementary Figure 1, https://stacks.cdc.gov/view/cdc/128424) varied by jurisdiction. For example, Alaska had one of the highest prevalences of reported emotional abuse (42.2%) but one of the lower prevalences of physical abuse (19.4%). Among jurisdictions that asked all eight types of ACE questions, the prevalence of adults reporting four or more ACEs ranged from 11.9% (New Jersey) to 22.7% (Oregon). Geographic patterns of reporting four or more ACEs also differed by age group (Supplementary Figure 2, https://stacks.cdc.gov/view/cdc/130206), with some consistent regional differences observed across age groups (e.g., increased prevalence of reporting 4 or more ACEs among jurisdictions in the Pacific Northwest).

## Discussion

This study provides the first estimates of ACEs among U.S. adults for all 50 states and the District of Columbia using BRFSS data. During 2011–2020, nearly two thirds of U.S. adults reported at least one ACE, and approximately one in six U.S. adults reported four or more ACEs. Among certain sociodemographic groups, for example, AI/AN or multiracial adults, these numbers are even higher, reflecting inequities in socioeconomic conditions that increase risk for ACEs. These numbers also highlight the potential intergenerational impact of ACEs through lost opportunities and lasting impacts on behavior and health (*8*). The prevalence of ACEs is strikingly lower among adults aged ≥65 years than among younger age groups; although this might be due to recall bias or differing trends over time, it might also reflect the risk of premature mortality accompanying exposure to a high number of ACEs (*9*).

Patterns in individual and total number of ACEs varied widely by jurisdiction and among sociodemographic groups, reinforcing the importance of population-level and local collection of ACE data to inform targeted prevention and intervention strategies. Variations in ACEs can result from several factors: differing demographic patterns, jurisdiction-level policies related to domestic violence, economic supports for families, historical and ongoing trauma because of discrimination, and social conditions (*4*). Better understanding of the relative contributions of these factors to ACEs in individual jurisdictions can help policymakers identify the most promising areas for intervention and the populations with the greatest need for services (*4*). Jurisdictions could consider further contextualizing their ACEs data with other BRFSS questions, such as those examining social determinants of health. CDC has released prevention resources to help provide jurisdictions and communities with the best available strategies to prevent violence and other ACEs, including guidance on how to implement those strategies for maximum impact (*4*–*6*). Clinicians and others who work directly with families play an important role in mitigating and preventing ACEs, from primary prevention opportunities (e.g., home visitation programs), to secondary and tertiary prevention strategies that reduce harms associated with ACEs (e.g., trauma-informed care, ensuring required linkage to services, and supports for identified issues) (*10*).

The findings in this report are subject to at least four limitations. First, data were collected over a 10-year period; prevalence might have changed in jurisdictions without recent data. In addition, jurisdiction-specific prevalences reflect the experiences of adults living in that jurisdiction, but do not necessarily represent the jurisdiction in which the ACE occurred. Second, although most jurisdictions used identical measures, two states (Arkansas and New Hampshire) collapsed or omitted sexual abuse questions, and one state (New Hampshire) omitted two types of ACEs. As a result, estimates for emotional abuse and parental separation or divorce are unavailable for New Hampshire. The reported prevalences of ACEs might be underestimated because respondents with missing ACEs data (79,797) were excluded from the analysis; these respondents reported higher prevalence of individual ACEs on the questions they did answer than those who answered all of the ACEs questions. Third, recall and social desirability biases might reduce the accuracy of self-reported ACEs, leading to underestimation, because participants might no longer remember or be willing to disclose potentially traumatic events from their childhood. Finally, BRFSS questions measure a limited set of ACEs and do not reflect the full range, severity, or frequency of ACEs. It is possible that ACEs included in BRFSS are experienced differently by certain groups, thereby shaping some of the demographic and geographic differences observed. In addition, certain limitations need to be considered when interpreting jurisdiction-specific estimates. First, BRFSS records a small subset of potential ACEs; there might be ACEs that are particularly relevant in certain parts of the country that are not included on BRFSS (e.g., experiences of racism or discrimination and community violence) and are thereby not reflected in estimates. Second, adults with six or more ACEs die approximately 20 years earlier on average than do those without ACEs (*9*); survivorship bias might undercount ACE prevalence in regions affected by premature mortality related to ACEs. Despite these limitations, the findings from this study update the baseline for ACEs measurement from previous estimates from 25 states (*1*), providing actionable data for all 50 states and the District of Columbia.

ACEs are common, but not equally distributed within the population. Differing patterns by jurisdiction and sociodemographic characteristics demonstrate the importance of collecting ACEs data at the jurisdiction level to understand the scope of the problem, identify populations more affected by ACEs, and ACEs-related outcomes; to help guide prevention and mitigation interventions and policies (*6*). CDC has released prevention resources to help provide jurisdictions and communities with the best available strategies to prevent violence and other ACEs, and with guidance on how to implement those strategies for maximum impact (*4*–*6*). Clinicians and others who work directly with families play an important role in mitigating and preventing ACEs, from primary prevention opportunities (e.g., home visitation programs) to secondary and tertiary prevention strategies that reduce harms associated with ACEs (e.g., trauma-informed care, ensuring appropriate linkage to services, and supports for identified issues) (*10*).

**Summary**

**What is already known about this topic?**

Adverse childhood experiences (ACEs) are associated with numerous negative outcomes. Previous data from 25 states indicated that ACEs are common among U.S. adults.

**What is added by this report?**

Among U.S. adults from all 50 states and the District of Columbia surveyed during 2011–2020, approximately two thirds reported at least one ACE; one in six reported four or more ACEs. ACEs were highest among women, persons aged 25–34 years, non-Hispanic American Indian or Alaska Native adults, non-Hispanic multiracial adults, adults with less than a high school education, and adults who were unemployed or unable to work. Prevalence of individual and total number of ACEs varied across jurisdictions.

**What are the implications for public health practice?**

CDC’s Preventing Adverse Childhood Experiences: Leveraging the Best Available Evidence provides strategies for preventing and mitigating ACEs, particularly among disproportionately affected populations.

## Footnotes

*
https://www.cdc.gov/violenceprevention/communicationresources/pub/technical-packages.html

†
https://www.cdc.gov/brfss/questionnaires/index.htm

§ Arkansas’ sexual abuse question was worded, “How often did anyone at least 5 years older than you or an adult ever touch you sexually, try to make you touch them sexually, or force you to have sex?” New Hampshire only included one of the three sexual abuse questions, “How often did anyone at least 5 years older than you or an adult ever touch you sexually?”

¶ Arkansas’ substance abuse question was worded, “Did you live with anyone who was a problem drinker or alcoholic, or who used illegal street drugs or abused prescription medications?”

** Generally, for ACE questions with response options of “Yes/No/Don’t know,” “Yes” was coded as experiencing the ACE, “No” was coded as not experiencing the ACE, and “Don’t know” was coded as missing. For ACEs questions with response options of “Never/Once/More than once/Don’t know,” “Never” was coded as not experiencing the ACE, “Once” or “More than once” was coded as experiencing the ACE, and “Don’t know” was coded as missing. For the substance use ACE, a “Yes” response to either the alcohol use or illegal drug or prescription drug misuse questions was coded as experiencing the substance use ACE. If the response to either alcohol use or illegal drug or prescription drug misuse questions was “No” and the other question response was missing, the substance use ACE was coded as missing. For the divorce or separation ACE, “Yes” was coded as experiencing the ACE, “No” was coded as not experiencing the ACE, and responses of “Parents not married” or “Don’t know” were coded as missing. For the sexual abuse ACE, three individual sexual abuse questions were combined to form a composite, dichotomous sexual abuse ACE. If answers to any of the sexual abuse questions was “Once” or “More than once,” the composite sexual abuse ACE was coded as experiencing the ACE. If answers to all of the sexual abuse questions was “Never,” the composite sexual abuse ACE was coded as not experiencing the ACE. If the respondent answered “Never” to one or more questions but was missing responses for one or more of the other sexual abuse questions, the response was coded as missing.

†† 45 C.F.R. part 46.102(I)(2), 21 C.F.R. part 56; 42 U.S.C. Sect. 241 (d); 5 U.S.C. Sect. 552a; 44 U.S.C. Sect. 3501 et seq.
